# Methionine Alkylation as an Approach to Quantify Methionine Oxidation Using Mass Spectrometry

**DOI:** 10.1021/jasms.3c00337

**Published:** 2024-02-07

**Authors:** Margaret Hoare, Ruiyue Tan, Kevin A. Welle, Kyle Swovick, Jennifer R. Hryhorenko, Sina Ghaemmaghami

**Affiliations:** † Department of Biology, University of Rochester, Rochester, New York 14627, United States; ‡ University of Rochester Mass Spectrometry Resource Laboratory, Rochester, New York 14627, United States

**Keywords:** methionine oxidation, methionine alkylation, proteomics, mass spectrometry (MS)

## Abstract

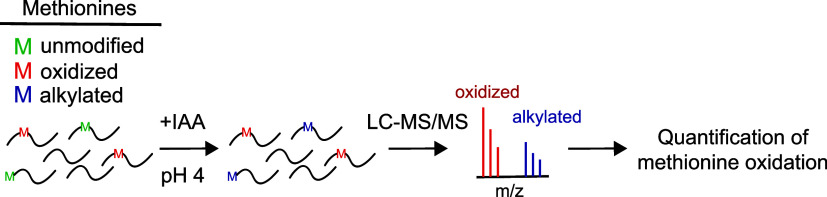

Post-translational oxidation of methionine residues can destabilize proteins or modify their functions. Although levels of methionine oxidation can provide important information regarding the structural integrity and regulation of proteins, their quantitation is often challenging as analytical procedures in and of themselves can artifactually oxidize methionines. Here, we develop a mass-spectrometry-based method called Methionine Oxidation by Blocking with Alkylation (MObBa) that quantifies methionine oxidation by selectively alkylating and blocking unoxidized methionines. Thus, alkylated methionines can be used as a stable proxy for unoxidized methionines. Using proof of concept experiments, we demonstrate that MObBa can be used to measure methionine oxidation levels within individual synthetic peptides and on proteome-wide scales. MObBa may provide a straightforward experimental strategy for mass spectrometric quantitation of methionine oxidation.

## Introduction

Methionine is a sulfur-containing amino acid that is susceptible to oxidation.^1,2^ Side chain oxidation converts nonpolar methionine residues to polar methionine sulfoxides, and this change in hydrophobicity can dramatically alter the structure and function of proteins.^3−7^ The conversion of methionine to methionine sulfoxide can occur chemically through reactions with reactive oxygen species (ROS) or enzymatically through the action of specific oxygenases.^8−11^ Observed levels of methionine oxidation *in vivo* are also influenced by cellular activities of a class of reducing enzymes known as methionine sulfoxide reductases (Msrs).^12^ As a post-translational modification, methionine oxidation has been implicated in protein damage, cellular signaling, ROS scavenging, pathological aging, and etiology of several neurodegenerative diseases.^6,13−15^

Techniques for quantifying methionine oxidation include FT-IR spectroscopy, chromatography, and mass spectrometry.^16−23^ Among these, methods involving tandem mass spectrometry (LC-MS/MS) are particularly advantageous, as they enable measurements of methionine oxidation on proteome-wide scales.^17,18,22,24^ For example, a protocol referred to as COFRADIC takes advantage of changes in liquid chromatography (LC) peptide retention times in bottom-up proteomic experiments to globally quantify methionine sulfoxide levels.^22,24^ However, a general complication with mass spectrometric analysis of methionine oxidation is the unpredictable accumulation of background oxidation that occurs during proteomic workflows, in particular during the process of electrospray ionization (ESI).^25,26^ Hence, because of methionines’ propensity for spurious oxidation, it is often difficult to unequivocally differentiate between methionine sulfoxides that form *in vivo* and those that artifactually accumulate during the subsequent mass spectrometric analyses.^18^

An alternative mass spectrometric approach that circumvents the artifactual oxidation of methionines is Methionine Oxidation by Blocking (MObB).^16−18,21^ In MObB, unoxidized methionines within peptides are forcibly oxidized with excess levels of ^18^O-labeled hydrogen peroxide, thus preventing further spontaneous ^16^O oxidation during mass spectrometric analysis. Accurate oxidation levels can subsequently be determined by measuring relative ratios of ^18^O- to ^16^O-modified peptide levels following a bottom-up proteomic workflow. Although MObB provides a straightforward proteomic approach for quantitation of methionine oxidation, it has a number of experimental complications. First, because ^18^O- and ^16^O-labeled peptides vary in mass by only 2 Da, their spectral isotopic envelopes are overlapped, and measuring their relative levels requires nonstandard data analysis procedures.^17,18^ Second, the quantitative accuracy of the method is contingent on the high isotopic purity of ^18^O-labeled hydrogen peroxide. Third, ^18^O-labeled hydrogen peroxide is a costly reagent that can be difficult to obtain. In fact, at the time of writing this manuscript, the previously available commercial sources for ^18^O-H_2_O_2_ (Sigma and Cambridge Isotope Laboratories) had discontinued its sale. Thus, it would be advantageous to develop an alternative method that can selectively block and quantify methionines using more standard and commonly available reagents. Here, we describe a protocol named Methionine Oxidation by Blocking with Alkylation (MObBa) for selective alkylation and quantitation of unoxidized methionines by iodoacetamide (IAA). In comparison to ^18^O-H_2_O_2,_ IAA is significantly less expensive, commonly available, and amenable to standard proteomic workflows and data analysis. We determine the precision of this technique and assess its ability to quantify methionine oxidation levels on proteome-wide scales.

## Results

### Methionine Can Be Fully Alkylated by Iodoacetamide at Low pH

Although the alkylation of cysteine thiols is a common practice in proteomic workflows, the alkylation of methionines has been comparatively less explored.^27−30^ It has long been known that methionines can be selectively alkylated at a pH of 2–5 with iodoacetate (IA) and iodoacetamide (IAA).^31−35^ More recently, conjugation of methionines with a range of other modifying reagents has also been described.^36,37^ The selective alkylation of unoxidized methionines, as detected by shifts in native gel electrophoretic mobility, has previously been used as an approach to quantify levels of oxidation in intact proteins.^31,35^ Here, we take advantage of the fact that methionines (but not methionine sulfoxides) can be alkylated at low pH to develop a method for quantitation of methionine oxidation in bottom-up proteomic experiments.

**Figure 1:**
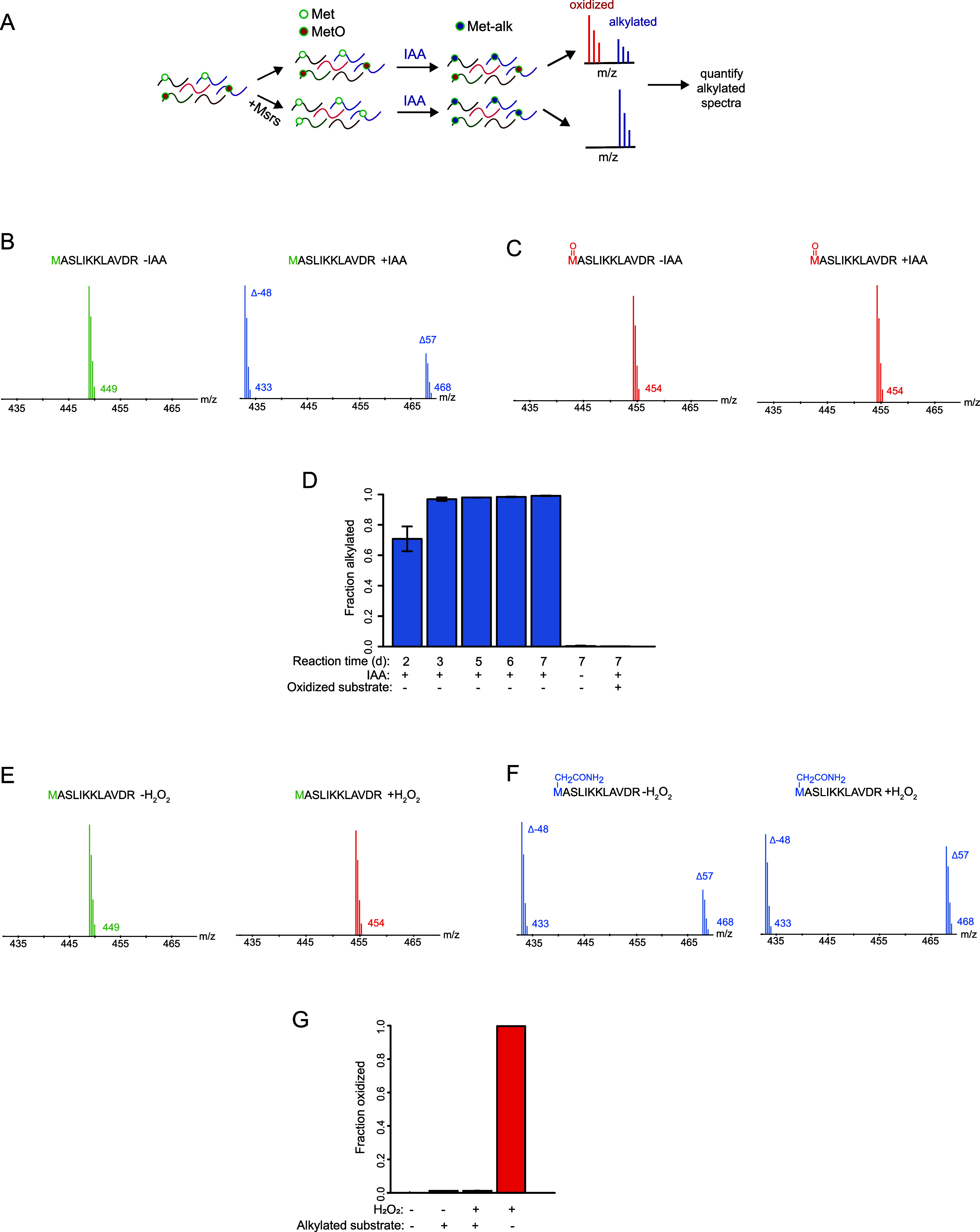
Unoxidized methionines can be fully alkylated by iodoacetamide. **(A)** The overall workflow of MObBa. Unoxidized methionines are selectively alkylated by iodoacetamide (IAA). This alkylation prevents further spurious oxidation and can be quantified by mass spectrometry as a proxy for the fraction of methionines that are unoxidized in the sample. **(B,C)** The synthetic peptide MASLIKKLAVDR can be fully alkylated by IAA, and this alkylation is inhibited by methionine oxidation. Unoxidized or oxidized peptides were used as substrates as indicated. In the experiments that generated the illustrated spectra, peptides were either treated with IAA at pH 4 for 7 days or left untreated. The green, blue, and red spectra indicate the expected *m*/*z* of the +3 charge state of the unoxidized, alkylated (including both Δ57 and Δ−48 modifications), and oxidized forms of the peptide, respectively. **(D)** The bar plot shows the time-course of the alkylation reaction. Fractional alkylation was quantified by adding the total intensities of the carbamidomethylation (Δ57) and dethiomethylation (Δ−48) modifications and dividing by the total intensity of the peptide. The error bars indicate the standard deviations of two replicate experiments. **(E,F)** The alkylation of the synthetic peptide MASLIKKLAVDR inhibits subsequent methionine oxidation. Alkylated or unalkylated peptides were used as substrates as indicated. For the experiments that generated the illustrated spectra, peptides were either treated with 160 mM H_2_O_2_ at pH 5 for 30 min or left untreated. The spectra are colored according to the scheme described in B,C. **(G)** The bar plot shows the quantitation of fractional oxidation as measured by dividing the intensity of the oxidized peptide by the total intensity. The error bars indicate the standard deviations of two replicate experiments.

In MObBa, polypeptides are treated with IAA at low pH to specifically modify unoxidized methionines and block downstream oxidation (Figure 1A). Fractional oxidation levels are then determined by measuring peptide alkylation levels relative to fully reduced Msr-treated controls. We initially set out to validate the chemical premise of MObBa using a methionine-containing synthetic peptide. Specifically, we verified that (1) methionines can be fully alkylated at low pH with IAA, (2) methionine oxidation inhibits its alkylation, and (3) methionine alkylation inhibits its oxidation. We carried out alkylation reactions at pH 4 over the course of 7 days on a synthetic methionine-containing peptide (MASLIKKLAVDR) (see Materials and Methods for details). The efficiency of alkylation was determined by mass spectrometry. The data indicate that methionine can be fully alkylated by IAA, and this alkylation is completely inhibited by oxidation (Figure 1B,C). The kinetics of this reaction at 37 °C is relatively slow, and full alkylation is achieved after ∼3 days (Figure 1D). The alkylation of the peptide completely inhibits its subsequent oxidation by H_2_O_2_, indicating that it is an effective blocking strategy (Figure 1E–G).

It had been previously reported that the alkylation of methionines by IAA yields two detectable modified peptide products in LC-MS/MS experiments.^28,30^ The first is a +57 Da product generated by carbamidomethylation of the side chain (Δ57), and the second is a −48 Da neutral loss product resulting from the alkylation-induced dethiomethylation of the side chain (Δ−48). Both of these products were detectable in the alkylated samples and together accounted for the entire population of modified peptides (Figure 1B,F).

### Methionine Alkylation by Iodoacetamide Is Selective

**Figure 2:**
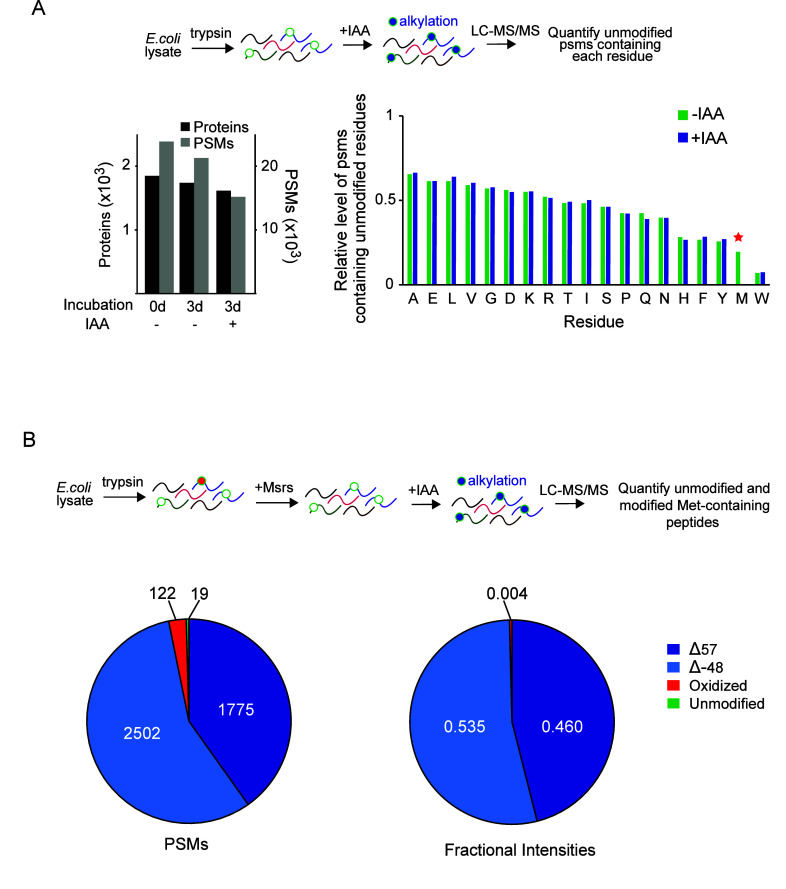
Methionine can be selectively alkylated by iodoacetamide in proteome-wide experiments. **(A)** Peptide mixtures generated by trypsinization of *E. coli* extracts were left untreated or incubated in the presence or absence of IAA at pH 4 for 3 days and analyzed by LC-MS/MS. The left histogram indicates the proteomic coverage of each experiment in terms of proteins and PSMs detected. The protein and PSM counts do not include peptides with alkylated methionines. In the right histogram, for the +IAA and −IAA incubated samples, numbers of PSMs containing each residue (in their unmodified forms) were divided by the number of all detected peptides to determine the relative population of each unmodified residue within the population. Cysteines were not included, as they are prealkylated prior to the low pH IAA treatment. **(B)** Tryptic peptides generated from *E. coli* extracts were treated with Msrs to remove background methionine oxidation and then incubated with IAA and analyzed by LC-MS/MS to determine the number of PSMs (left) and fractional total intensities (right) for methionine-containing peptides harboring only one methionine in their unmodified, dethiomethylated (Δ−48), carbamidomethylated (Δ57), and oxidized forms.

We next determined whether methionine can be selectively alkylated without artifactual modification of non-sulfur-containing residues. An unoxidized peptide mixture was generated from an *E. coli* protein extract by reducing disulfide bonds, alkylating free cysteines with IAA at neutral pH, and digesting the sample with trypsin. The sample was then treated with IAA at low pH for 3 days. As cysteines were already fully alkylated, they were not further modified by this treatment. Treated samples were analyzed by LC-MS/MS and searched against the *E. coli* proteome. Levels of modification for each amino acid were determined by quantifying the number of PSMs harboring the unmodified form of each residue (Figure 2A). The data indicated that IAA almost fully depleted peptides containing unmodified methionines, while relative levels of peptides containing unmodified forms of other residues were largely unaffected. In this search, less than 20 PSMs with N-terminal or lysine alkylation were detected. In a second experiment, the peptide mixture was initially reduced by treatment with Msrs and then treated with IAA as above. LC-MS/MS analysis indicated that nearly all identified methionine-containing peptides in IAA-treated samples harbored either methionine carbamidomethylation (Δ57) or dethiomethylation (Δ−48) (Figure 2B).

### MObBa Accurately Measures Protein Oxidation Levels

**Figure 3:**
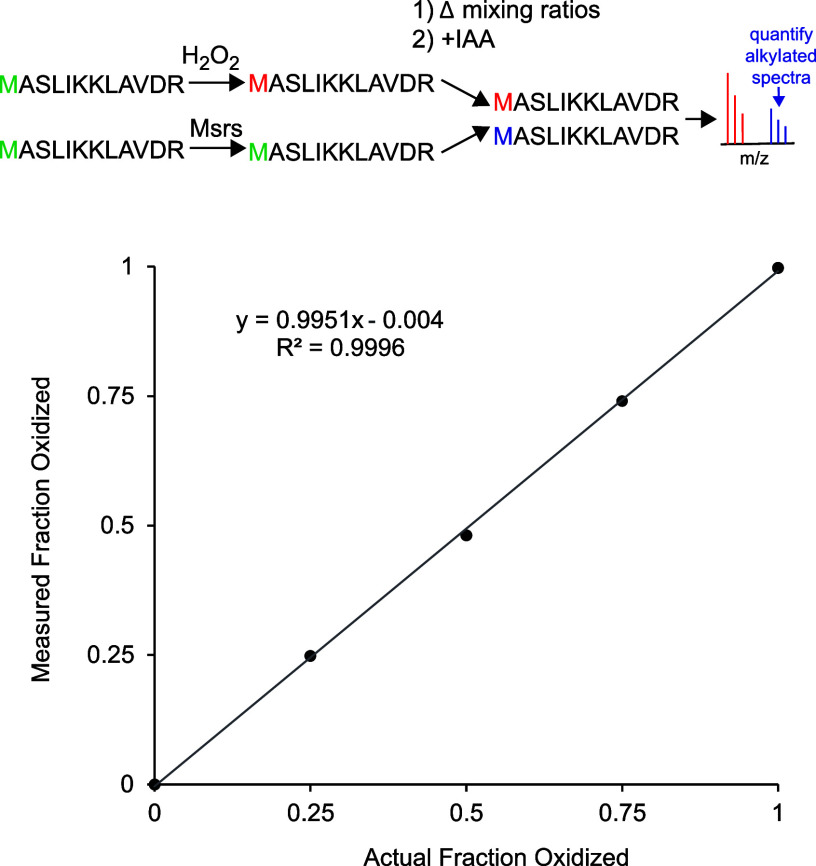
MObBa can accurately quantify methionine oxidation levels for a synthetic peptide. The synthetic peptide MASLIKKLAVDR was exposed to two treatments: full oxidation of methionine with hydrogen peroxide and full reduction of methionine oxidation by Msrs. Mixtures of the two treatments (containing 0, 25, 50, 75, 100% oxidized peptides) were incubated with IAA and analyzed by mass spectrometry. For each mixture, fractional oxidation was measured by calculating the intensities of alkylated peptides after IAA treatment and normalizing with respect to the 0% oxidized samples. The error bars indicate the standard deviations of two technical replicate experiments.

To demonstrate the quantitative accuracy of MObBa, we generated and analyzed peptide mixtures with known levels of methionine oxidation. A methionine-containing synthetic peptide was fully oxidized by the addition of H_2_O_2_ or completely reduced by addition of Msrs (Figure 3). The two samples were mixed at variable ratios to obtain different known levels of methionine oxidation. The mixtures were then treated with IAA, and oxidation levels were quantified by summing the intensities of Δ57 and Δ−48 modified peptides. The results indicated a high level of quantitative accuracy and a linear response across the range of analyzed fractional oxidation levels.

**Figure 4:**
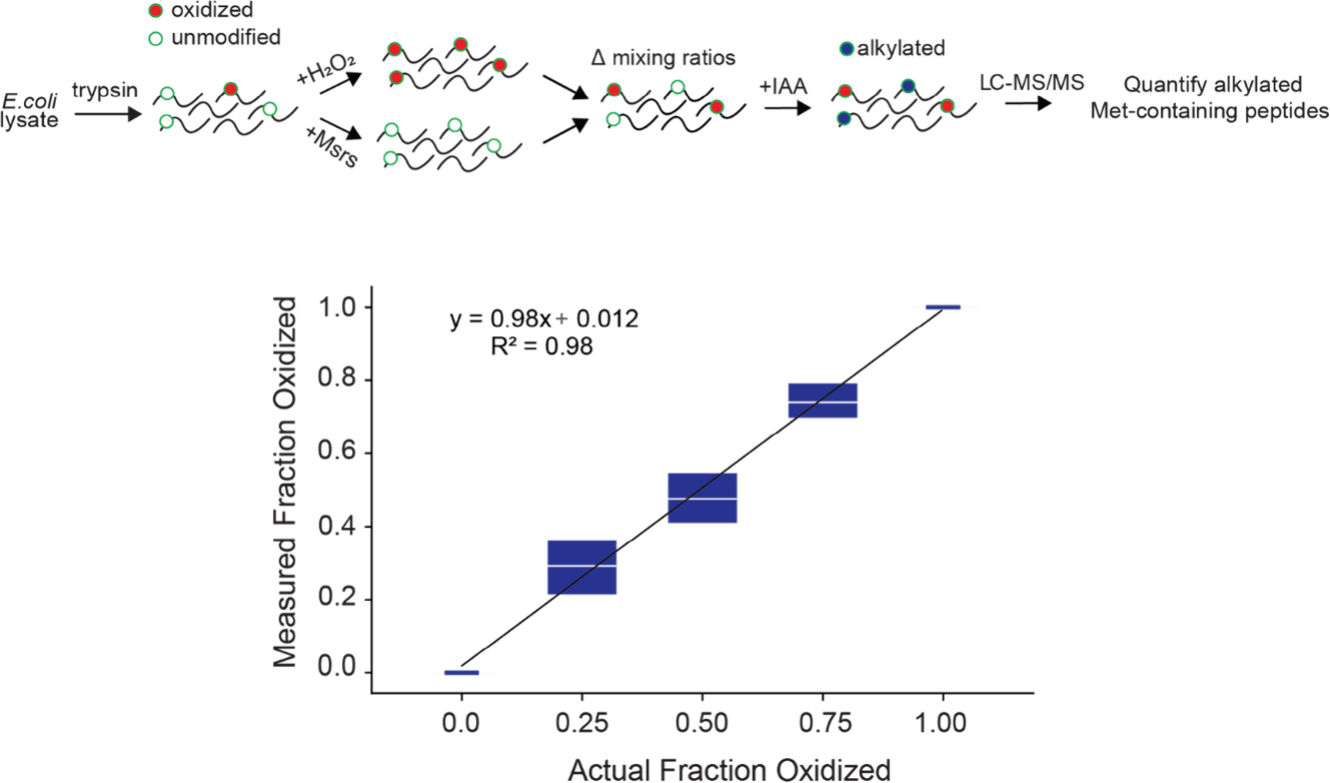
MObBa accurately measures methionine oxidation in proteome-wide experiments. Tryptic peptides derived from *E. coli* were exposed to two treatments: full oxidation by hydrogen peroxide and full reduction by Msrs. Mixtures of the two treatments (containing 0, 25, 50, 75, and 100% oxidized peptides) were incubated with IAA and analyzed by LC-MS/MS. Fractional oxidation of each detected peptide was measured as in Figure 3. The box plot indicates the interquartile distribution of the measurements in each condition, and the white line indicates the median.

We next repeated the above experiment on the entire *E. coli* proteome (Figure 4). Proteins were extracted from *E. coli* and digested into peptides by trypsin after reduction and blocking of cysteines. The peptide mixture was divided into two different treatment conditions: (1) full reduction of oxidized methionines by Msrs and (2) full oxidation of methionines by hydrogen peroxide. Peptides from these two conditions were mixed to generate specific fractional oxidation levels. Each mixture was then treated with IAA at low pH, analyzed by LC-MS/MS, and searched against the *E. coli* proteome using the Δ57 and Δ−48 alkylated forms as variable modifications. Fractional oxidation levels were measured by normalizing the intensities of alkylated peptides relative to the unoxidized control. The results indicated that the distribution and median oxidation measurements of peptides corresponded to expected levels within each mixture.

## Discussion

An important consideration for accurate quantitation of methionine oxidation by mass spectrometry is the prevention of artifactual oxidation that occurs during the sample preparation and ionization steps of typical proteomics workflows. In this study, we show that treatment of peptides with IAA at low pH alkylates unoxidized methionines and prevents their subsequent oxidation during mass spectrometric analysis. Thus, quantitation of alkylated methionines following IAA treatment provides an effective metric for levels of oxidized methionines that were present prior to alkylation.

The experiments presented here validate MObBa as an approach for quantifying methionine oxidation in both isolated polypeptides and complex peptide mixtures. In our proof of concept experiments, we treated trypsinized extracts with methionine sulfoxide reductases to reduce any endogenous oxidation and then mixed the unoxidized proteomes with fully oxidized proteomes to generate different predetermined oxidation stoichiometries that were then experimentally measured by MObBa. In future experiments, analysis of unreduced cell extracts by this approach may enable the measurement of endogenous methionine oxidation levels within cells.

However, our experiments also highlight important caveats that must be considered when implementing this strategy. First, the alkylation of methionines is a relatively slow reaction, occurring over the course of 3 days at 37 °C. The slow reaction rate not only adds to the overall analysis time but may also lead to accumulation of oxidation and other modifications prior to alkylation. In the experiments described above, we minimized this effect through careful degassing of all samples, conducting reactions under nitrogen gas and normalizing alkylation levels with respect to fully reduced control samples. These precautions significantly reduced background oxidation levels and the long incubation periods in and of themselves did not significantly reduce proteomic coverage (Figure 2). Second, peptide ions harboring carbamidomethylated methionines appear to be unstable in electrospray mass spectrometry and result in a neutral loss product where the methionine side chain is dethiomethylated. Thus, to account for all blocked peptide products, both carbamidomethylated and dethiomethylated modified forms of methionine must be included in database searches. The side chain neutral loss also prohibits the possible use of isotopically labeled IAA as an approach to compare alkylation levels across samples.^38^ However, as an alternative, it may be possible to combine MObBa with metabolic labeling (e.g., SILAC) to more accurately measure differences in oxidation levels between peptide samples obtained from cells exposed to different treatment conditions. Third, unlike MObB where measurements of methionine oxidation levels within individual samples are internally normalized by comparison of ^16^O- to ^18^O-labeled spectra, MObBa is reliant on comparison of alkylation levels across runs and thus is potentially subject to higher levels of experimental error.

Although the above-mentioned limitations may limit the use of MObBa in some proteomic experiments, the methodology offers a number of advantages compared to alternative methods for quantitation of methionine oxidation. It is a straightforward protocol involving IAA, a commonly used reagent that is part of typical bottom-up proteomics workflows. Analysis of data generated by MObBa does not require specialized software and can be conducted using commonly available search algorithms. Thus, MObBa may prove useful for analyses that require quantitation of methionine oxidation without requiring unconventional procedures and reagents.

## Conclusion

Treatment of peptide samples with IAA at low pH selectively alkylates unoxidized methionines and provides a strategy for quantitation of methionine oxidation in bottom-up proteomic experiments. In proof of concept experiments, the applicability and quantitative accuracy of the approach was demonstrated for individual synthetic peptides and complex peptide mixtures. In comparison to existing methodologies, the advantages of this approach include its ease of implementation within typical bottom-up proteomic workflows and lack of requirement for specialized and expensive reagents. However, to effectively employ this strategy, a number of factors must be taken into account including the slow rate of the alkylation reaction and the generation of neutral loss products.

## Materials and Methods

### Peptide Preparation

The synthetic peptide MASLIKKLAVDR was purchased from GenScript at 97.7% purity. The *E. coli* peptide extract was prepared from an *E. coli* K12 W3110 strain. Cells were grown in LB media at 37 °C, pelleted, then lysed in 5% SDS in 50 mM TEAB through sonication at 25 A. Cellular debris were pelleted by centrifugation at 16 000*g* for 15 min. Protein concentrations were quantified from the supernatant using a bicinchoninic acid (BCA) kit (Thermo Scientific). 25 μg of protein was reduced with 2 mM dithiothreitol (Sigma) for 60 min at 55 °C. Cysteines were alkylated with 10 mM IAA (Sigma) for 30 min at room temperature in the dark. The alkylation reaction was quenched with 1.2% phosphoric acid. 90% methanol in 100 mM TEAB was added to the extract (6:1,v/v), and the sample was loaded onto S-trap micro filters (ProtiFi). The peptides were isolated in the filters and then digested with 1 μg of trypsin (Pierce) and incubated overnight at 37 °C in a water bath. After incubation, the filters were centrifuged for 1 min at 4000*g* and then eluted with 40 μL of 0.1% trifluoroacetic acid (TFA) in H_2_O (Thermo Scientific) and 40 μL of 50% acetonitrile (ACN)/H_2_O in 0.1% TFA (Thermo Scientific). The peptides were lyophilized and reconstituted in degassed 5% formic acid (FA) in H_2_O.

### Alkylation of Peptide-Bound Methionines

For the synthetic peptide, 15 μg of peptide was incubated with 50 μL of 33 mM IAA in degassed 5% FA (pH 4) under nitrogen gas at 37 °C. Unless otherwise stated, the reaction was carried out for 3 days. The samples were then desalted in a homemade C18 column and eluted in 50% ACN/H_2_O in 0.1% formic acid. *E. coli* peptide extracts were alkylated as above after reconstitution in 5% FA.

### Oxidation of Peptide-Bound Methionines

For both synthetic peptides and *E. coli* extracts, 25 μg of peptide was oxidized with 50 μL of 160 mM H_2_O_2_ for 30 min at 37 °C. The sample was then frozen and lyophilized to remove excess hydrogen peroxide, then desalted in a homemade C18 column and eluted in 50% ACN/H_2_O in 0.1% formic acid.

### Expression and Purification of MsrA and MsrB

pET 151/D-TOPO expression vectors coding for *E. coli* MsrA and MsrB containing N-terminal His tags downstream of a T7 promoter were synthesized (Invitrogen GeneArt) and transformed into BL21(DE3) competent cells (Thermo Scientific) by heat shock. Cells were plated and placed under ampicillin selection overnight at 37 °C. A 10 mL culture of LB media was inoculated with single colony and grown to an OD_600_ of 0.6–0.8. The culture was subsequently added to 1 L of LB media and induced with 400 μM IPTG, then incubated overnight at 25 °C while shaking at 180 rpm. The bacteria were pelleted by centrifugation at 8000*g* for 3 min. The cells were placed in PBS buffer containing 20 mM imidazole and EDTA free protease inhibitor at pH 7.4 and sonicated. The lysate was centrifuged at 6000*g* for 30 min at 4 °C, and the pellet was discarded. A nickel column was made with Ni-NTA resin (Thermo Scientific) and equilibrated with two washes of 20 mM sodium phosphate and 10 mM imidazole in PBS at pH 7.4. The lysate was run through the column by gravity flow. The column was then washed with 25 mM imidazole at pH 7.4 until no protein was detected in the flowthrough by UV absorption. Bound proteins were eluted with 250 mM imidazole in pH 7.4. Dialysis was performed to transfer proteins into degassed 50 mM Tris buffer at pH 7.4. Protein concentrations were determined by BCA assay. The final purity of the MsrA and MsrB enzymes were ∼91 and ∼94%, respectively, as determined by SDS-PAGE. The activity of the purified enzymes was verified by measuring reduction of oxidized peptides as detected by mass spectrometry (see below).

### Reduction of Peptide-Bound Methionine Sulfoxides by Msrs

For the synthetic peptide, 25 μg of the peptide was incubated with 50 mM DTT, 1.5 μg of MsrA, and 5.0 μg of MsrB in 50 mM Tris buffer at pH 7.4 for 45 min at 37 °C. Samples were lyophilized and then desalted to remove enzymes and salts. *E. coli* peptide extracts were reduced as above.

### Mass Spectrometric Analysis

Synthetic peptides were diluted to 15 μg/mL in 50% ACN/H_2_O in 0.1% FA. 50 μL of the sample was run in a Q Exactive Plus Mass Spectrometer (Thermo Scientific) by direct injection with a Dionex Ultimate 3000 with a flow rate of 100 μL/min for 3 min. The solvent consisted of a 50% mixture of 0.1% FA in H_2_O and 0.1% FA in ACN. Peptides were ionized by a HESI source set in positive mode. Data were collected over a range of 300–2000 *m*/*z* at a resolution of 70K at *m*/*z* 200 with a 240 ms maximum injection time and AGC target of 1e6.

*For E. coli* extracts, peptides were injected onto a 75 μm × 2 cm trap column prior to refocusing on a homemade 100 μm × 15 cm C18 column with 1.8 μm beads (Sepax), using a Vanquish Neo UHPLC (Thermo Fisher), connected to an Orbitrap Astral mass spectrometer (Thermo Fisher). Solvent A was 0.1% formic acid in water, while solvent B was 0.1% formic acid in 80% acetonitrile. Ions were introduced to the mass spectrometer using a Nanospray Flex source operating at 2 kV. The gradient began at 1% B and ramped to 5% B in 0.1 min, increased to 30% B in 9.8 min, increased to 40% in 0.7 min, and finally increased to 99% B in 0.1 min and was held for 3.3 min to wash the column for a total runtime of 14 min. After each run was completed, the column was re-equilibrated with 1% B prior to the next injection. Due to the fact that we were looking for several different modifications, the Orbitrap Astral was operated in data-dependent mode, with MS1 scans acquired in the Orbitrap and MS2 scans acquired in the Astral analyzer. The 100 most abundant peaks during each cycle were selected for fragmentation. Monoisotopic Precursor Selection (MIPS) was set to Peptide. The full scan was done over a range of 375–1400 *m*/*z*, with a resolution of 120 000 at *m*/*z* of 200, AGC Target set to Standard, and a maximum injection time of 5 ms. Only peptides with a charge state between 2 and 5 could be picked for fragmentation. Precursor ions were fragmented by higher energy collisional dissociation (HCD) using a collision energy of 27% with an isolation width of 2 *m*/*z*, a maximum injection time of 5 ms, and a normalized AGC target of 200%. Dynamic exclusion was set to 10 s.

### Measurement of Fractional Oxidation

For synthetic peptide experiments, raw MS data were analyzed by the XCalibur software (Thermo Scientific). The total intensity of the peptides containing the alkylation modifications was summed, and fractional alkylation was calculated in the experimental sample. For Figures 1 and 3, MS1 spectra were exported using the MSConvert software, and intensities of alkylated or oxidized peaks were measured using Mathematica (Wolfram) and RStudio. Fractional alkylation was measured by summing the total intensities of the carbamidomethylated and dethiomethylated forms of the peptide and normalizing this intensity relative to the 0% oxidized Msr-treated samples. Fractional oxidation was determined by subtracting the fractional alkylation from 1.

For proteome-wide experiments, raw data were searched using the SEQUEST search engine within the Proteome Discoverer software platform, version 3.1 (Thermo Fisher), using the Uniprot *Escherichia coli* database. Trypsin was selected as the enzyme allowing up to two missed cleavages, with an MS1 mass tolerance of 10 ppm and an MS2 mass tolerance of 0.02 Da. Carbamidomethyl cysteine was set as a fixed modification, while oxidation, carbamidomethylation, and dethiomethylation on methionine were set as variable modifications. Percolator was used as the FDR calculator, filtering out peptides which had a q-value greater than 0.01. The oxidation level analyses were conducted using the measured PSM intensities as described in the Results section. Fractional alkylation levels were calculated using the precursor abundance intensities for the carbamidomethylated and dethiomethylated versions of a peptide in the preoxidized sample divided by the precursor abundance intensities for the carbamidomethylated and dethiomethylated versions of the peptide in the 0% oxidized (Msr-treated) samples. Proteome Discoverer by default uses the intensity of the highest peak of the isotopic envelope for each peptide at the apex of the chromatographic profile for quantitation. However, analysis of a representative set of PSMs indicated that similar measurements are obtained through analysis of spectral peak areas.

Raw spectra and search results for all experiments have been deposited in the PRIDE database (Acc: PXD045497).

## Data Availability

All raw and processed data are available at the ProteomeXchange Consortium via the PRIDE partner repository (accession number PXD045497).

## Abbreviations

ACN: acetonitrile
BCA: bicinchoninic acid
ESI: electrospray ionization
FA: formic acid
MObBa: Methionine Oxidation by Blocking with Alkylation
ROS: reactive oxygen species
Msrs: methionine sulfoxide reductases
MObB: Methionine Oxidation by Blocking
LC-MS/MS: liquid chromatography tandem mass spectrometry
LC: liquid chromatography
IAA: iodoacetamide
IA: iodoacetate
Δ57: methionine carbamidomethylation
Δ−48: methionine dethiomethylation
SILAC: Stable Isotopic Labeling by Amino Acids in Cell Culture
TFA: trifluoroacetic acid
DDA: data-dependent acquisition
MIPS: Monoisotopic Precursor Selection
CID: collision-induced dissociation
